# Donor-Transmitted Cancer in Orthotopic Solid Organ Transplant Recipients: A Systematic Review

**DOI:** 10.3389/ti.2021.10092

**Published:** 2022-02-04

**Authors:** George H. B. Greenhall, Maria Ibrahim, Utkarsh Dutta, Carolyn Doree, Susan J. Brunskill, Rachel J. Johnson, Laurie A. Tomlinson, Chris J. Callaghan, Christopher J. E. Watson

**Affiliations:** ^1^ Department of Statistics and Clinical Research, NHS Blood and Transplant, Bristol, United Kingdom; ^2^ School of Immunology and Microbial Sciences, King’s College London, London, United Kingdom; ^3^ GKT School of Medical Education, King’s College London, London, United Kingdom; ^4^ Systematic Review Initiative, NHS Blood and Transplant, Oxford, United Kingdom; ^5^ Department of Non-Communicable Disease Epidemiology, London School of Hygiene and Tropical Medicine, London, United Kingdom; ^6^ Department of Nephrology and Transplantation, Guy’s Hospital, Guy’s and St Thomas’ NHS Foundation Trust, London, United Kingdom; ^7^ Department of Surgery, University of Cambridge, Cambridge, United Kingdom; ^8^ The Cambridge NIHR Biomedical Research Centre, Cambridge, United Kingdom; ^9^ NIHR Blood and Transplant Research Unit in Organ Donation and Transplantation, University of Cambridge, Cambridge, United Kingdom

**Keywords:** liver transplantation, cancer, heart transplantation, lung transplantation, donor-transmitted disease, deceased organ donors

## Abstract

Donor-transmitted cancer (DTC) has major implications for the affected patient as well as other recipients of organs from the same donor. Unlike heterotopic transplant recipients, there may be limited treatment options for orthotopic transplant recipients with DTC. We systematically reviewed the evidence on DTC in orthotopic solid organ transplant recipients (SOTRs). We searched MEDLINE, EMBASE, PubMed, Scopus, and Web of Science in January 2020. We included cases where the outcome was reported and excluded donor-derived cancers. We assessed study quality using published checklists. Our domains of interest were presentation, time to diagnosis, cancer extent, management, and survival. There were 73 DTC cases in liver (n = 51), heart (n = 10), lung (n = 10) and multi-organ (n = 2) recipients from 58 publications. Study quality was variable. Median time to diagnosis was 8 months; 42% were widespread at diagnosis. Of 13 cases that underwent re-transplantation, three tumours recurred. Mortality was 75%; median survival 7 months. Survival was worst in transmitted melanoma and central nervous system tumours. The prognosis of DTC in orthotopic SOTRs is poor. Although re-transplantation offers the best chance of cure, some tumours still recur. Publication bias and clinical heterogeneity limit the available evidence. From our findings, we suggest refinements to clinical practice.

**Systematic Review Registration:** https://www.crd.york.ac.uk/prospero/display_record.php?ID=CRD42020165001, Prospero Registration Number: CRD42020165001.

## Introduction

Donor-transmitted cancer (DTC) occurs when a tumour is transferred from an organ donor to the recipient via the transplanted organ. Improvements in cancer care and an ageing population have led to an increase in the proportion of donors with a history of cancer, which may put more solid organ transplant recipients (SOTRs) at risk of DTC (1–4).

A diagnosis of DTC has major implications. Survival is often poor and treatment options may be limited (5–8). The optimal treatment in heterotopic SOTRs (e.g., kidney or pancreas transplant recipients) usually comprises discontinuation of immunosuppression followed by allograft removal. This can lead to cancer remission, even in cases with widespread dissemination (9, 10). However, this option is not readily available to orthotopic SOTRs (e.g., heart, lung, liver recipients), so these patients and their clinicians face difficult decisions and significant uncertainty. A transmission event also has implications for other recipients of organs from the same donor, who may consider pre-emptive re-transplantation. Previous reviews in this area have shown variable outcomes in SOTRs with DTC. However, these included recipients with donor-derived cancer (DDC), which results from neoplastic transformation of donor cells following transplantation and often has different treatment implications for affected patients (11, 12). There are no reviews of DTC across all types of orthotopic SOTRs. Guidance on surveillance or treatment of SOTRs with or at risk of DTC is lacking (4, 13–15).

Given the paucity of information on DTC in orthoptic SOTRs, we systematically reviewed the published literature in this area. Our review addressed the following questions: (1) how and when does DTC present in orthoptic SOTRs? (2) what treatment strategies have been used? (3) what are the outcomes after treatment, including re-transplantation? We aimed to synthesise the available evidence in this area in order to suggest refinements to clinical practice.

## Materials and Methods 

We undertook a prospectively registered systematic review (PROSPERO ID CRD42020165001) (16). We followed the PRISMA and “Synthesis Without Meta-analysis” guidelines for study reporting (17, 18).

### Inclusion and Exclusion Criteria

Our review population was orthotopic SOTRs with DTC. In accordance with published guidelines, we defined DTC as a cancer of donor origin in an SOTR, which was known or assumed to be present in the donor at the time of transplantation (8). Importantly, we excluded cases of DDC. Studies were eligible if they described recipients of liver, heart, lung, or intestinal transplants with DTC, and reported transplant type, transmitted cancer type, presentation or management, and patient survival (i.e., vital status at the time of reporting). We included any publication type except review articles and editorials.

### Search Strategy

We searched MEDLINE (1946 to present), EMBASE (1974 to present), PubMed (e-publications ahead of print only), Scopus, and Web of Science Core Collection. Our search terms included “cancer,” “tumour,” “transplant,” “donor,” “transmission,” and all related terms. We limited our search to human studies but did not apply date or language restrictions. We used publicly available search filters to restrict our search to cohort studies, case-control studies, case series and case reports, because we did not expect to find any interventional studies (19). We then searched “grey” literature sources including non-indexed conference proceedings, thesis repositories, and the World Health Organisation “NOTIFY” library (20). Lastly, we hand-searched reference lists of included articles. We executed our search on January 16, 2020. Our full search strategy is in the Supplementary Material.

### Study Selection and Quality Grading

Two reviewers (GG, MI) independently screened titles and abstracts followed by full-text review to determine study eligibility. We resolved disagreements by discussion. Where cancer origin was unclear (DTC vs. DDC) we involved a senior author (CW) or contacted authors for clarification. We cross-checked all included cases to identify duplicates between publications and included the report with the most complete information on each case.

Two reviewers (GG, MI) independently scored the quality of each included study using tools published by the Joanna Briggs Institute (JBI), with resolution of disagreements by discussion (21, 22). These are tools designed to assess the methodological quality of a study objectively, using categorical responses (yes/no/unclear/not applicable) to questions on key domains (e.g., “was the current clinical condition of the patient on presentation clearly described?”). We assessed case reports and registry studies against the JBI checklists for case reports and prevalence studies, respectively. We did not exclude any studies on the basis of quality. Because we did not find any reports with a comparator group, we were unable to assess the risk of bias.

### Data Extraction

Two reviewers (GG, UD) independently extracted data from included studies using a pre-piloted proforma (see Supplementary Material), creating a separate record for each case included in our review. Our five main domains of interest were: mode of presentation, time to diagnosis, tumour extent, treatment, and survival time.

We recorded the publication type, year, and total number of DTC cases (including heterotopic transplants) in each article. We considered reports of multiple transplants from a single donor as case reports. For each DTC case, we recorded recipient demographics, transplant type, mode of presentation (symptoms, graft dysfunction, surveillance, *post-mortem*), time to diagnosis, primary tumour site and histology, and cancer extent at diagnosis (confined to allograft/distant metastases). Donor variables were: demographics, history of cancer, and time from cancer diagnosis to donation. Information on management comprised cancer-specific treatment (e.g., chemotherapy, radiotherapy, loco-regional therapy, tumour resection), re-transplantation (and time from diagnosis), and modification of immunosuppression. We recorded all time intervals in days if less than 1 month and in whole months if more than 1 month.

Our outcomes were patient death, cause of death, cancer remission and cancer recurrence (and time since remission). Unless stated otherwise, we assumed that treatment procedures with curative intent (re-transplantation, resection) achieved remission. Where articles reported death only, we assumed that remission was not achieved. Where information on these outcomes was missing from case reports, we contacted study authors by email.

### Statistical Analysis

We first examined data completeness across our main domains of interest (mode of presentation, time to diagnosis, tumour extent, treatment, survival time). We then tabulated donor, recipient, and tumour-related characteristics of all included cases. After analysing all cases, we stratified our dataset, first by transplant type and then by cancer type. We did this because both domains are relevant to scenarios encountered in clinical practice. We grouped cancer type by the site of the primary tumour (e.g., lung), unless we found only one histological type in a particular site (e.g., melanoma). We did not group our data by study type because we analysed information at individual case level.

We determined the range, median and interquartile range (IQR) of the time from transplantation to DTC diagnosis. We then calculated the proportion of tumours with spread beyond the allograft at diagnosis. We compared tumour extent between transplant types using the chi-squared test. We tabulated the treatment modalities reported. Among cases that received a second allograft, we determined the median time from diagnosis to re-transplantation. We then calculated the proportion of cases that achieved cancer remission. Among these, we summarised the treatment modalities received, the proportion that recurred, and the proportion that died.

Our main outcome was all-cause mortality, calculated as the proportion of cases that died after a diagnosis of DTC. Since we only included cases where survival was reported, the denominator here was all cases (or all within a group). We used all-cause mortality because some treatment modalities (e.g., re-transplantation) confer substantial risk, so this is the most relevant patient-related outcome. The lack of comparator groups in each study precluded meta-analysis of treatment effects. Due to the size and heterogeneity of our study, multivariable analyses were not appropriate (23).

To analyse survival time, we restricted our dataset to cases with follow-up of at least 6 months, or to death. We assessed the heterogeneity of cases included in this analysis by summarising the range of follow-up time. We determined the median survival time following DTC diagnosis in all cases, then stratified by transplant and cancer type (for the commonest cancers).

*Post hoc*, we explored factors that may influence survival among cases with sufficient follow-up (6 months, or to death). Due to substantial variation in follow-up between studies, we censored this analysis at 3 years from DTC diagnosis. We examined the relationship between survival time and (1) transplant type (2), tumour extent at diagnosis, and (3) re-transplantation, using Kaplan-Meier curves and log-rank tests. To reduce confounding, we restricted our analysis of re-transplantation to cases with tumour confined to the allograft at diagnosis. We did this because patients with disseminated cancer would not ordinarily be considered suitable for re-transplantation, making them an inappropriate comparator group. Owing to data sparsity, it was not appropriate to test for an association between cancer type and survival time. Lastly, we tallied the number of cases of DTC in heterotopic SOTRs that received organs from the same donors as our included cases, and the proportion that died.

We performed study screening with Covidence software (Veritas Health Innovation, Australia), data extraction with EpiData v4.6 (EpiData Association, Denmark), and data analysis with Stata v15.1 (StataCorp, United States).

## Results

### Study Selection

**FIGURE 1:**
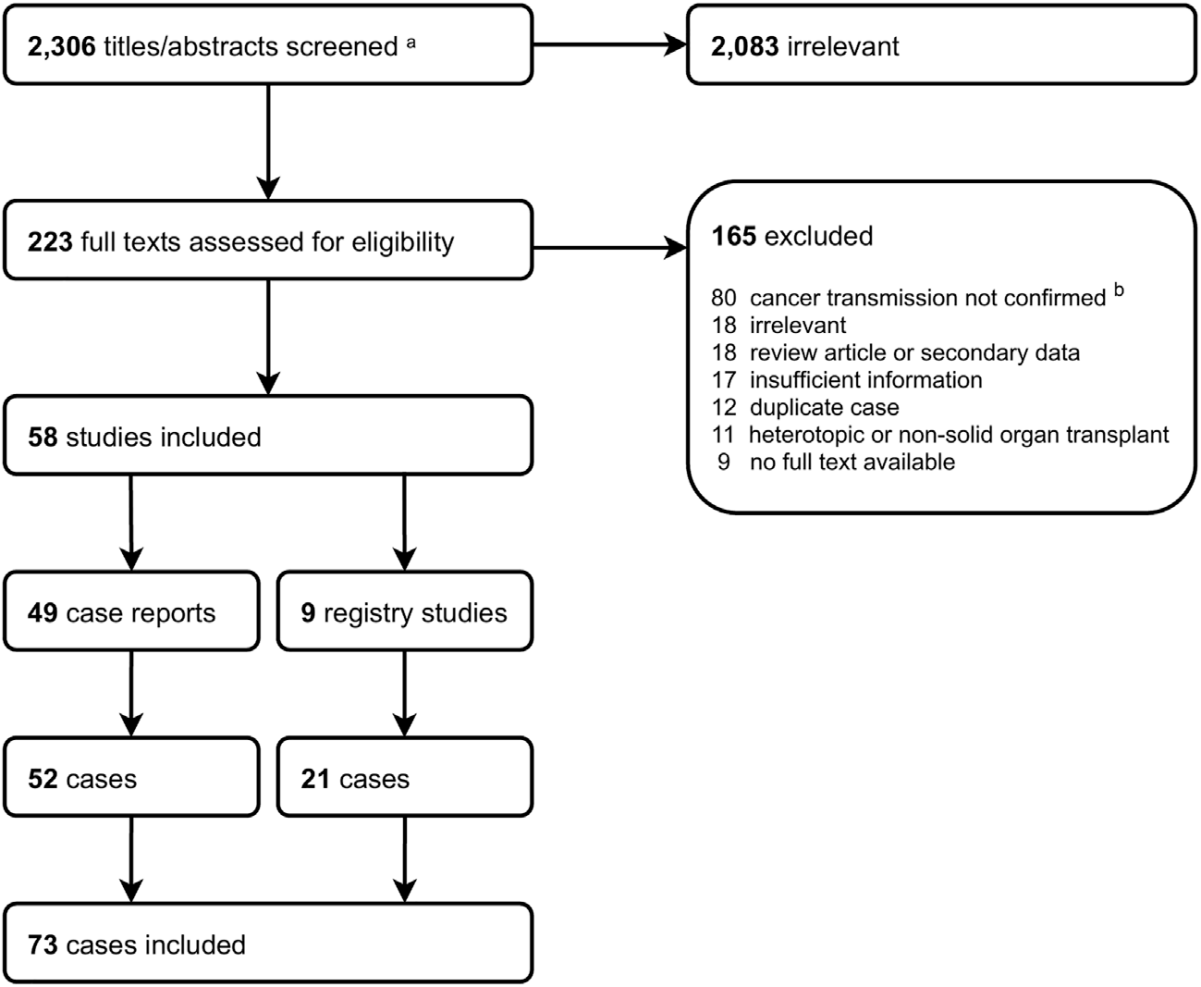
Study screening flowchart. ^a^Includes 2,298 from search strategy and 8 from hand searches (5 from WHO NOTIFY Library, 3 from reference lists). ^b^Includes donor-derived cancer.

Our search retrieved 2,308 articles. After title and abstract screening, we assessed 223 full texts against our inclusion criteria. Fifty-eight articles (49 case reports, 9 registry studies) published between 1987 and 2019 were eligible for inclusion (Figure 1). Our review population comprised 73 cases of DTC in orthotopic SOTRs (52 from case reports and 21 from registry studies). These 73 cases originated from 69 donors and were reported from North America (n = 37), Europe (n = 33), Asia (n = 1), Australia (n = 1) and South America (n = 1). Supplementary Table S1 shows the characteristics of all included studies.

### Study Quality

Overall, the quality of included articles was acceptable. However, there was substantial variation between studies and across quality domains. Among case reports, the domains with the lowest quality were the clinical condition of the patient at presentation and after treatment. The quality of registry studies was lower; most provided insufficient information on study size, case identification methods and sample coverage. Supplementary Figure S1, and Supplementary Figure S2 summarise study quality scoring against the JBI checklists.

Among the 73 cases in our study, data completeness varied across our five domains of interest (Supplementary Table S2). The proportion of cases with information in each domain was: tumour extent, 84% (61/73); time to diagnosis, 89% (65/73); presentation, 73% (50/73); treatment, 67% (49/73); survival time, 60% (44/73). We contacted the study authors of eight cases with incomplete outcome data; four replied with supplementary information which was added to the dataset for analysis.

### DTC Presentation

Table 1 summarises the characteristics of included cases. There were 52 liver (including one liver-intestine-pancreas transplant), 10 heart, and 11 lung recipients (including one heart-lung transplant). Median (IQR) recipient age at diagnosis was 53 (41–60) years; 51% (37/73) were male. Median (IQR) donor age was 50 (39–62) years. In 29/73 (40%) cases, a cancer had been found in the donor. Six of these were diagnosed before donor assessment (between 4 months and 32 years prior to death), while 23 were discovered after organ implantation.

**TABLE 1:** Characteristics of cases of donor-transmitted cancer included in review, by transplant type

Total cases	Organ transplanted			All cases
	Liver^a^	Heart	Lung^b^	
	52	10	11	73
Tumour identified in donor	16 (31%)	7 (70%)	6 (55%)	29 (40%)
Time to cancer diagnosis (months)	8 (4–12)	10 (5–12)	9 (3–14)	8 (4–12)
Tumour spread beyond allograft at diagnosis	14 (27%)	10 (100%)	7 (64%)	31 (42%)
Re-transplanted	13 (25%)	0	0	13 (18%)
Survival after DTC diagnosis (months)^c^	9 (2–36)	6 (3–23)	2 (1–5.5)	7 (2–31)

a includes 1 liver-intestine-pancreas.

b includes 1 heart-lung.

c restricted to cases with follow-up of at least 6 months, or to death (n = 49; 36 liver, 5 heart, 8 lung).

Numbers are n (%) or median (IQR).

Table 2 summarises the types of transmitted malignancies included in our study. The commonest histological types were melanoma (n = 10) and choriocarcinoma (n = 7). Supplementary Table S3 shows the histology of all included cases. The most frequent mode of presentation was with symptoms, in 24/73 (33%) cases. Other methods of case detection were surveillance imaging (either routine or targeted because of transmission risk, n = 14), graft dysfunction (n = 4), tumour markers (elevated β-human chorionic gonadotropin in transmitted choriocarcinoma, n = 3), or retrieval or implantation biopsy (n = 5). Four cases were diagnosed at recipient *post-mortem* only.

**TABLE 2:** Characteristics of cases of donor-transmitted cancer included in review, by primary cancer type.

Primary tumour	Cases	Transplant type (n)	Time to diagnosis (m)	Spread beyond allograft at diagnosis	Re-transplanted	Died
Melanoma	10	Liver (6), Heart (2), Lung (2)	11 (9–13)	6	0	10/10
Choriocarcinoma	7	Liver (5), Heart (2)	1.5 (1–3)	7	0	5/7
CNS tumours	7	Liver (4),^a^ Heart (1), Lung (2)	4.5 (4–9)	5	0	7/7
Genitourinary tumours	7	Liver (3), Heart (2), Lung (2)^b^	11 (9–14)	4	1	5/7
Haematological malignancies	7	Liver (6), Heart (1)	12 (1–18)	3	1	6/7
Neuroendocrine tumours^c^	7	Liver (7)	9 (8–36)	1	2	4/7
Lung tumours	6	Liver (3), Heart (1), Lung (2)	6 (4–9)	2	1	5/6
Sarcomas	6	Liver (4), Lung (2)	2.5 (1–8)	1	1	4/6
Tumours of unknown primary site	6	Liver (5), Heart (1)	6 (6–12)	1	3	3/6
Intestinal tumours	5	Liver (5)	11 (6–13)	0	2	4/5
Other tumours^d^	5	Liver (4), Lung (1)	5 (0–16)	1	2	2/5

a includes 1 liver-pancreas-intestine transplant.

b includes 1 heart-lung transplant.

c includes 1 small cell neuroendocrine tumour of lung origin.

d breast (2), hepatocellular (2), pancreas (1).

Numbers are n or median (IQR); m, months. See Supplementary Table S2 for full histological details of cases included.

CNS, central nervous system.

Time from transplantation to DTC diagnosis ranged from 0 days to 6 years. In total, 48/73 (66%) cases were diagnosed within 1 year, and 60/73 (82%) within 2 years. Median (IQR) time to diagnosis was 8 (4–12) months; this was similar across transplant types (Table 1). The cancer types with the shortest time to diagnosis were choriocarcinoma [median (IQR) 1.5 (1 to 3) months] and sarcoma [2.5 (1 to 8) months; Table 2].

At the time of diagnosis, 29/73 (40%) tumours were confined to the allograft while 31/73 (42%) had disseminated. Twelve cases (eight heart, two liver, one heart-lung) had distant metastases only, with no tumour in the allograft. There was strong evidence of an association between tumour extent and transplant type; 27% (14/52), 100% (10/10) and 64% (7/11) of liver, heart, and lung recipients, respectively, had tumour dissemination at diagnosis (χ^2^ = 15.2, *p* = 0.001; Table 1). It also varied between cancer types; all cases of choriocarcinoma had spread beyond the allograft at diagnosis, whereas all intestinal tumours and 6/7 neuroendocrine tumours (NETs) were confined to the allograft (Table 2).

### DTC Management

Excluding palliative management, 43/73 (59%) cases included treatment details (Table 3). The commonest treatment was systemic chemotherapy; this was used in 20 cases and was the main treatment in 14. Seven cases underwent tumour resection and seven received loco-regional therapies, comprising radio/chemo-embolisation (n = 3), radiofrequency ablation (n = 2), brachytherapy (n = 1), and extracorporeal proton therapy (n = 1). There were six reports of altered immunosuppressive regimens, comprising a switch from calcineurin inhibitors to sirolimus (n = 4) or everolimus (n = 2).

**TABLE 3:** Treatment modalities for cases of donor-transmitted cancer included in review.

	Cases
Total cases with treatment reported	43
Cancer treatment	
Chemotherapy^a^	20 (47%)
Tumour resection^b^	7 (16%)
Loco-regional therapy^c^	7 (16%)
External beam radiotherapy	6 (14%)
Immunosuppression management	
Reduction	14 (33%)
Cessation	3 (7%)
Drug change^d^	6 (14%)
Re-transplantation	13 (30%)

a includes 1 patient treated with chemotherapy and hormone therapy for prostate cancer.

b excludes re-transplantation.

c radio/chemo-embolisation (3), radiofrequency ablation (2), brachytherapy (1), extracorporeal proton therapy (1).

d calcineurin inhibitor switch to sirolimus (4) or everolimus (2).

Numbers are n (%). Some cases received more than one treatment. Excludes cases with only palliative management.

Thirteen cases underwent re-transplantation. All of these were liver recipients that had no tumour dissemination at diagnosis, including one case pre-emptively re-transplanted after a *post-mortem* donor cancer diagnosis (DTC from the first donor subsequently recurred) (24). Re-transplantation was performed at a median (IQR) of 4 months (4 days–6 months) following DTC diagnosis. Treatments received prior to re-transplantation were loco-regional therapy (n = 3), tumour resection (n = 1), and chemotherapy (n = 1). Following re-transplantation, 3/13 (23%) tumours recurred between 2 weeks and 3 years later, and three patients died (Table 4).

**TABLE 4:** Cases of donor-transmitted cancer undergoing re-transplantation (all liver recipients).

Transmitted cancer (References)	Time from transplantation to diagnosis	Time from diagnosis to re-transplantation	Cancer recurrence (time from re-transplantation)	Died	Total follow-up^a^ (months)
NET (29)	8 months	5 months	Yes (17 days)^b^	Yes	9
NET (30)	36 months	24 months	No	No	36
Colonic adenocarcinoma (31)	13 months	9 months	No	No	33
Colonic adenocarcinoma (32)	4 months	4 months^c^	No	Yes^d^	40
Lung adenocarcinoma (24)	11 months	-^e^	Yes (11 months)	Yes	13
Urothelial tumour (33)	14 months	7 days	No	No	48
Sarcoma (34)	0 days	4 days	No	No	76
Plasmacytoma (35)	0 days	9 days	Yes (36 months)	No	42
Pancreatic adenocarcinoma (36)	0 days	1 days	No	No	12
HCC (37)	1 days	3 days	No	No	36
SCC, unknown primary (38)	6 m	6 m	No	No	6
Adenocarcinoma, unknown primary (39)	12 m	NR	No	No	8
Adenocarcinoma, unknown primary (40)	6 months	6 months	No	No	31

a from DTC diagnosis.

b pancreatic metastases found 2 weeks following re-transplantation; cancer remission not achieved.

c initially resected, subsequently re-transplanted.

d died of pneumonia.

e pre-emptive re-transplantation on day 7 after donor cancer found at autopsy—recurrence 11 months later.

SCC, squamous cell carcinoma; NET, neuroendocrine tumour; NR not reported; HCC, hepatocellular carcinoma.

In total, 19/73 (26%) cases achieved cancer remission following treatment. The main treatment modalities in these cases were: re-transplantation (n = 12; one case with recurrence 2 weeks after re-transplantation was not considered to have achieved remission), tumour resection (without subsequent re-transplantation, n = 5), loco-regional therapy alone (n = 1), and chemotherapy alone (n = 1). Of the 19 cases with cancer remission, six (33%) subsequently experienced a recurrence between 10 months and 3 years later, and five (26%) died (three of which had recurrent cancer).

### DTC Outcomes

In total, 55/73 cases (75%) died. This includes four cases diagnosed at *post-mortem*. Forty-seven deaths were due to cancer, three were due to other causes (sepsis, pneumonia, variceal bleed), and in five cases the cause of death was not evident. All-cause mortality was 69% (36/52), 80% (8/10), and 100% (11/11) in liver, heart, and lung recipients, respectively. Mortality by cancer type ranged from 50% in tumours of unknown origin to 100% in melanoma and central nervous system (CNS) tumours (Table 2).

**FIGURE 2:**
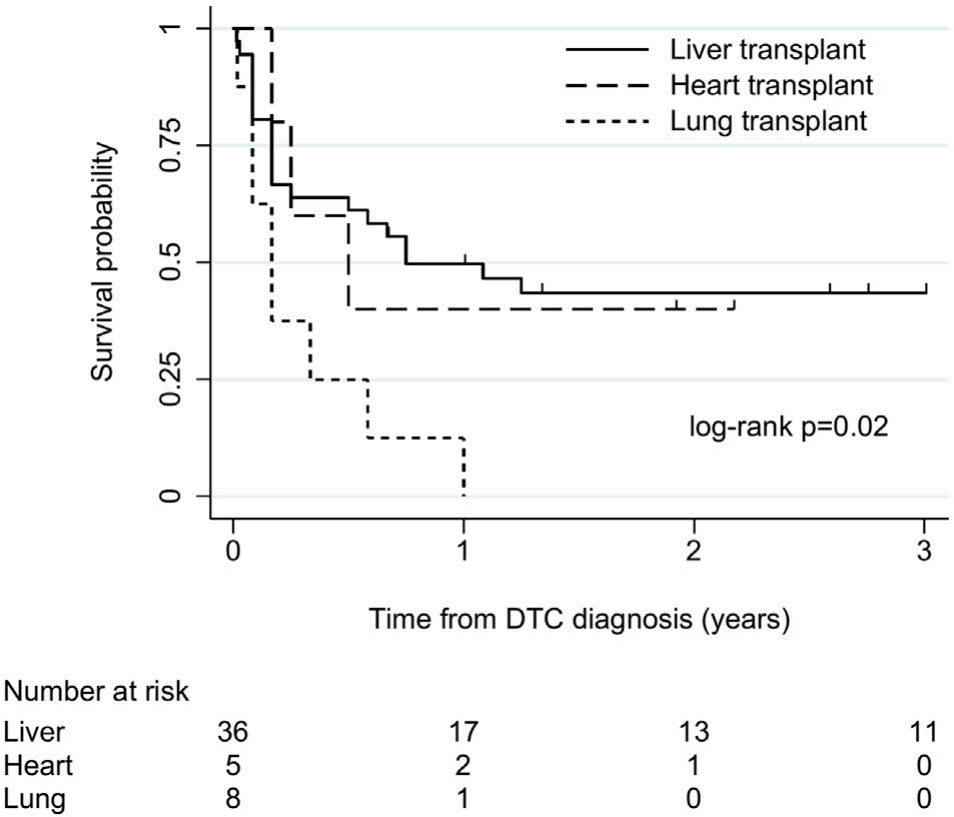
Kaplan-Meier plot of patient survival after donor-transmitted cancer diagnosis, by transplant type. Restricted to cases with follow-up of at least 6 months, or to death (n = 49). Follow-up censored at 3 years. Liver includes liver-pancreas-intestine (1), lung includes heart-lung (1). DTC, donor-transmitted cancer.

There were 49 cases (36 liver, 5 heart, 8 lung) with follow-up of at least 6 months or to death. Among these, survival after DTC diagnosis ranged from 5 days to 13 years. Overall, 1-year survival was 39% (19/49). Overall median (IQR) survival was 7 (2–31) months and 9 (2–36), 6 (3–23) and 2 (1–5.5) months in liver, heart, and lung recipients, respectively. There was some evidence of an association between transplant type and survival time (log-rank χ^2^ 8.3, *p* = 0.02; Figure 2), with the shortest survival in lung recipients. Survival time varied between the commonest cancer types. Median (IQR) survival was 2 (1–7) months in melanoma, 2 (1–2) months in CNS tumours, 9 (3–36) months in NETs and 26 (2–48) months in genitourinary tumours.

Median (IQR) survival was 16 (7–37) months in tumours confined to the allograft (n = 25) and 2 (1–9) months in disseminated cancers (n = 22). There was strong evidence of shorter survival in cases with tumour dissemination at diagnosis (log-rank χ^2^ 9.9, *p* = 0.002; Supplementary Figure S3).

**FIGURE 3:**
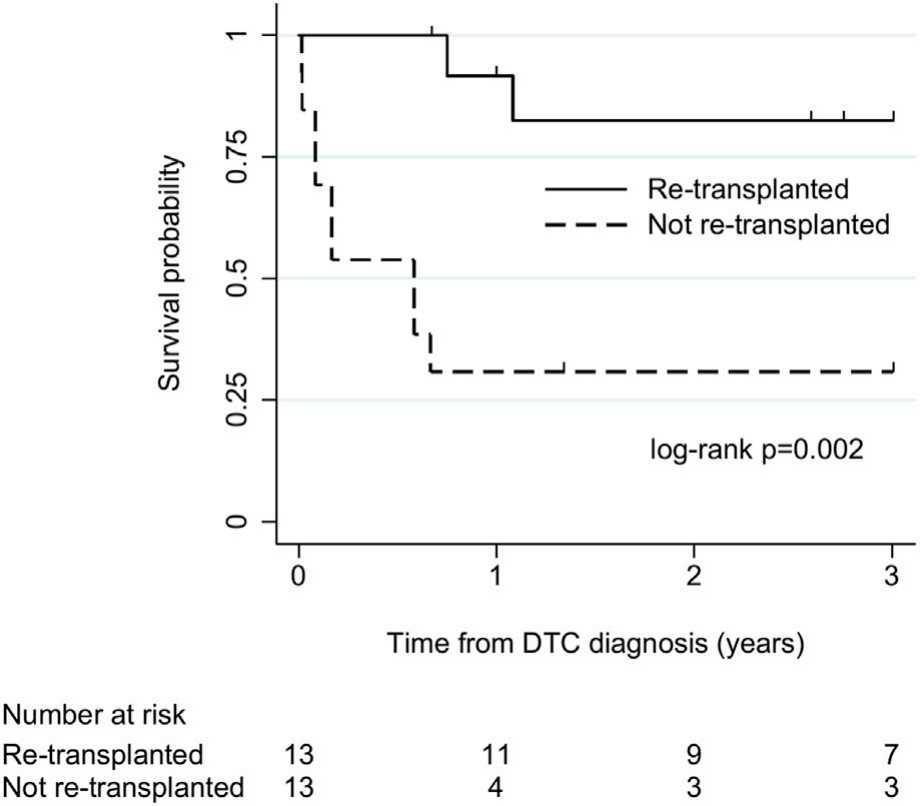
Kaplan-Meier plot of patient survival after donor-transmitted cancer diagnosis, by re-transplantation. Restricted to cases with follow-up of at least 6 months, or to death, and no tumour beyond allograft at diagnosis (n = 26). Follow-up censored at 3 years. Re-transplanted cases: liver recipients (n = 13); cases not re-transplanted: liver (n = 10), lung (n = 3) recipients. DTC, donor-transmitted cancer.

There were 26 cases (23 liver, 3 lung) without tumour dissemination at diagnosis and with sufficient follow-up for survival analysis. Among these, 13 (all liver) underwent re-transplantation, and 13 (10 liver, 3 lung) did not. All-cause mortality was 23% (3/13) in re-transplanted cases and 85% (11/13) in cases that were not re-transplanted. Median (IQR) survival was 36 (13–40) months and 7 (1–16) months in cases that did and did not undergo re-transplantation, respectively. There was strong evidence of longer survival in re-transplanted cases (log-rank χ^2^ 9.3, *p* = 0.002; Figure 3).

There were 44 cases of DTC in recipients of heterotopic transplants from the donors of the cases included in our review (42 kidney, 1 pancreas, 1 kidney-pancreas); 21 (48%) of these died.

## Discussion

In this systematic review of confirmed cases of donor-transmitted malignancies, we identified 73 orthotopic SOTRs with DTC in the published literature. The commonest malignancies were melanoma and choriocarcinoma. Most presented within 2 years of transplantation and nearly half had spread beyond the allograft at the time of diagnosis. Mortality was high: three-quarters died overall, 60% within a year.

Some characteristics varied between the cancer types that we identified. Choriocarcinoma appeared to be the most aggressive tumour, with early presentation and dissemination at diagnosis in all cases. Conversely, intestinal tumours presented later and were all confined to the allograft. We found the worst outcomes in melanoma, CNS tumours and haematological malignancies. Unsurprisingly, tumour dissemination at diagnosis conferred shorter survival.

We also found some variation between transplant types. Compared to heart and lung recipients, liver recipients were less likely to have tumour beyond the allograft at diagnosis and survived longer. This might be due to the lower level of immunosuppression that these patients require, or their suitability for re-transplantation; all re-transplanted cases in our review were liver recipients, and these had substantially better survival. Tumours recurred in nearly a quarter of re-transplanted cases.

Our study contains fewer cases than previous reviews in this area (11, 12). There are two main reasons for this. The first is our minimum data set for inclusion, which excluded cases with less detail reported. As a result, most cases in our review are from case reports, since most registry studies contain minimal individual-level clinical details. Second, we excluded cases of DDC. We did this because DTC is theoretically preventable, usually has a narrow window of presentation, and often has implications for other recipients of organs from the same donor, whereas DDC tends to present later and may have more favourable outcomes (5). The most striking consequence of this is in relation to lymphoma. We identified four recipients with donor-transmitted lymphoma, all of whom died, contrasting sharply with the 80% survival in 30 cases in a previous review of liver recipients (11). Although the origin of donor-related post-transplant lymphoproliferative disorders (PTLD) is controversial, outcomes in donor-derived PTLD may in fact be better compared to cancers of recipient origin (25, 26). Although our inclusion criteria focussed this review on one patient group, we acknowledge that differentiating between DTC and DDC is subjective; some excluded cases could have influenced our results.

Taken in the context of existing research, our study confirms that the prognosis of DTC in orthotopic SOTRs is worse than in heterotopic transplant recipients (12, 27). The outcomes of the heterotopic SOTRs with DTC from the same donors as our included cases appear to confirm this. However, this is most likely to reflect the optimal treatment strategy—cessation of immunosuppression, allograft removal and systemic anti-cancer therapy—which is available to heterotopic SOTRs, confounding any direct comparison with orthotopic SOTRs.

This is the first study to summarise the experience of DTC across all orthotopic SOTRs and compare outcomes between transplant types. We specifically examined the rate of cancer remission and recurrence, which have not been studied previously. We followed a prospectively registered protocol and identified cases according to international criteria. We took all possible steps to exclude duplicate cases from our review. There were several duplicated reports in the published literature. However, we cannot completely eliminate the possibility that some duplicates remain. Our study grading tools provided objective measures of quality and our minimum dataset for inclusion gave acceptable data completeness.

Our review confirms that the quality of evidence in this area is generally low. Published data are largely limited to anecdotal reports. This is unlikely to change. By definition, these studies do not include comparator groups, so it is difficult to judge the impact of patient or treatment factors in each study reliably. The most important limitation of the available evidence is publication bias. This limits direct comparisons between cases. It would be inappropriate to infer that the risk of transmission mirrors the frequency of cases in our study. Similarly, cases with a favourable outcome are more likely to be reported, which could bias our results; actual outcomes may be worse than our results suggest. Even compulsory reporting of transmission events, as mandated by many national transplant authorities, is prone to under-recognition or biased reporting. Registry linkage is one method to minimise biased case detection and outcome reporting.

There is also a significant amount of clinical heterogeneity in the published evidence. We acknowledge that treatments reported were chosen on a case-by-case basis and may have been published for their novelty, limiting interpretation of our findings. Variable follow-up may have biased our survival analyses. Although we mitigated this by restricting our analyses to cases with sufficient follow-up (at least 6 months) and censoring follow-up at a reasonable point, selection bias remains likely. Assessing survival from the time of DTC diagnosis may also have introduced immortal time bias. The wide time span of publications in our review meant that we could not account for temporal changes in therapeutic options.

The size of our review population limits the power of our analyses; this is inevitable with such a rare condition. Anticipating this, we avoided multivariable analyses which could have introduced more uncertainty. This means we were unable to address the possibility of other factors confounding our results. Grouping cancers by primary site resulted in significant heterogeneity within some groups; histological type may be a more important determinant of tumour behaviour in the host environment. Our main outcome (all-cause mortality) may have been vulnerable to bias because the review population were likely to be at increased risk of death from other causes (e.g., infection), as a result of immunosuppressive therapy, complications of organ failure, or anti-cancer treatment. However, since most deaths were due to cancer, this is unlikely to have changed our findings meaningfully. There was also a certain amount of missing data.

Within the limits of the evidence base, we feel it is reasonable to make some suggestions for practice improvement. Firstly, our findings support surveillance of orthotopic SOTRs at increased risk of DTC for at least 2 years, because approximately 80% of cases present during this time. We suggest this applies to recipients of transplants from donors with tumours that have more than a “minimal” transmission risk, as defined by international guidelines (14, 15, 28), or those notified of a transmission event from their donor. Tumour characteristics should dictate the type of surveillance; imaging or laboratory studies may be more appropriate. It is notable that one in six cases in our review presented without tumour in the allograft, meriting careful consideration of surveillance imaging. Secondly, monitoring for allograft dysfunction does not appear to be a reliable means of detecting DTC in this population, since only a minority of the cases included in our review presented in this way. Thirdly, if a transmitted tumour is confined to an orthotopic allograft, re-transplantation should be considered. However, the physiological state of the recipient and the availability of a suitable organ will influence this decision since it carries substantial morbidity. Tumour resection or loco-regional therapy may achieve remission while avoiding a second major operation; further experience of these treatments in the context of DTC will benefit the transplant community. There are other important knowledge gaps, including the role of tumour markers in donor assessment or recipient surveillance, optimal management of immunosuppression before/after re-transplantation, and longer-term outcomes in re-transplanted patients.

In summary, this review confirms the poor prognosis of DTC in orthotopic SOTRs. Re-transplantation appears to offer the best hope of survival, but some tumours recur despite this. Further studies using prospectively collected data and disease registry linkage could shed more light on the diagnosis and treatment of this condition and inform guidance on surveillance of patients at risk.

## Abbreviations

CI, confidence interval; CNS, central nervous system; DDC, donor-derived cancer; DTC, donor-transmitted cancer; HCC, hepatocellular carcinoma; IQR, interquartile range; JBI, Joanna Briggs Institute; NET, neuroendocrine tumour; NR, not reported; PTLD, post-transplant lymphoproliferative disorder; SCC, squamous cell carcinoma; SD, standard deviation; SOTR, solid organ transplant recipient.

## References

[1] Garrido G Matesanz R. The Spanish National Transplant Organization (ONT) Tumor Registry. Transplantation (2008) 85(8 Suppl. l):S61–3. 10.1097/TP.0b013e31816c2f55 18425039

[2] Fiaschetti P Pretagostini R Stabile D Peritore D Oliveti A Gabbrielli F The Use of Neoplastic Donors to Increase the Donor Pool. Transplant Proc (2012) 44(7):1848–50. 10.1016/j.transproceed.2012.06.030 22974853

[3] Watson CJE Roberts R Wright KA Greenberg DC Rous BA Brown CH How Safe Is it to Transplant Organs from Deceased Donors with Primary Intracranial Malignancy? an Analysis of UK Registry Data. Am J Transpl (2010) 10(6):1437–44. 10.1111/j.1600-6143.2010.03130.x 20486904

[4] Kaul DR Vece G Blumberg E La Hoz RM Ison MG Green M Ten Years of Donor-Derived Disease: A Report of the Disease Transmission Advisory Committee. Am J Transpl (2020) 21:689–702. 10.1111/ajt.16178 32627325

[5] Myron Kauffman H McBride MA Cherikh WS Spain PC Marks WH Roza AM. Transplant Tumor Registry: Donor Related Malignancies. Transplantation (2002) 74(3):358–62. 10.1097/00007890-200208150-00011 12177614

[6] Desai R Collett D Watson CJ Johnson P Evans T Neuberger J. Cancer Transmission from Organ Donors-Unavoidable but Low Risk. Transplantation (2012) 94(12):1200–7. 10.1097/tp.0b013e318272df41 23269448

[7] Buell JF Trofe J Hanaway MJ Lo A Rosengard B Rilo H Transmission of Donor Cancer into Cardiothoracic Transplant Recipients. Surgery (2001) 130(4):660–8. 10.1067/msy.2001.117102 11602897

[8] Ison MG Nalesnik MA. An Update on Donor-Derived Disease Transmission in Organ Transplantation. Am J Transpl (2011) 11(6):1123–30. 10.1111/j.1600-6143.2011.03493.x 21443676

[9] Wilson RE Hager EB Hampers CL Corson JM Merrill JP Murray JE. Immunologic Rejection of Human Cancer Transplanted with a Renal Allograft. N Engl J Med (1968) 278(9):479–83. 10.1056/nejm196802292780904 4866045

[10] Zukoski CF Killen DA Ginn E Matter B Lucas DO Seigler HF. Transplanted Carcinoma in an Immunosuppressed Patient. Transplantation (1970) 9(1):71–4. 10.1097/00007890-197001000-00021 4904755

[11] Eccher A Girolami I Marletta S Brunelli M Carraro A Montin U Donor-Transmitted Cancers in Transplanted Livers: Analysis of Clinical Outcomes. Liver Transpl (2020) 27:55–66. 10.1002/lt.25858 32746498

[12] Xiao D Craig JC Chapman JR Dominguez-Gil B Tong A Wong G. Donor Cancer Transmission in Kidney Transplantation: a Systematic Review. Am J Transpl (2013) 13(10):2645–52. 10.1111/ajt.12430 24034231

[13] European Committee on Organ Transplantation. Guide to the Quality and Safety of Organs for Transplantation (2018). Available at: https://www.edqm.eu/en/organs-tissues-and-cells-technical-guides (Accessed July 2021).

[14] Advisory Committee on the Safety of Blood Tissues and Organs. Transplantation of Organs from Deceased Donors with Cancer. (2020) Available at: https://www.gov.uk/government/publications/transplantation-of-organs-from-donors-with-a-history-of-cancer (Accessed July, 2021).

[15] Nalesnik MA Woodle ES Dimaio JM Vasudev B Teperman LW Covington S Donor-transmitted Malignancies in Organ Transplantation: Assessment of Clinical Risk. Am J Transpl (2011) 11(6):1140–7. 10.1111/j.1600-6143.2011.03565.x 21645251

[16] Greenhall GHB Brunskill S Doree C Ibrahim M Callaghan C Watson CJE. Donor-transmitted Cancer in Orthotopic Solid Organ Transplantation: A Systematic Review. York, UK: PROSPERO (2020). Available at: https://www.crd.york.ac.uk/prospero/display_record.php?ID=CRD42020165001 . 10.3389/ti.2021.10092 PMC8842379 35185366

[17] Campbell M McKenzie JE Sowden A Katikireddi SV Brennan SE Ellis S Synthesis without Meta-Analysis (SWiM) in Systematic Reviews: Reporting Guideline. BMJ (2020) 368:l6890. 10.1136/bmj.l6890 31948937 PMC7190266

[18] Page MJ McKenzie JE Bossuyt PM Boutron I Hoffmann TC Mulrow CD The PRISMA 2020 Statement: an Updated Guideline for Reporting Systematic Reviews. BMJ (2021) 372:n71. 10.1136/bmj.n71 33782057 PMC8005924

[19] BMJ Best Practice. Study Design Search Filters. Available at: https://bestpractice.bmj.com/info/us/toolkit/learn-ebm/study-design-search-filters/ (Accessed January 2020).

[20] World Health Organisation. NOTIFY Library: The Global Vigilance and Surveillance Database for Medical Products of Human Origin. Available at: https://notifylibrary.org/ (Accessed January 2020).

[21] Moola S Munn Z Tufanaru C Aromataris E Sears K Sfetc R Chapter 7: Systematic Reviews of Etiology and Risk. Adelaide, Australia: JBI Manual for Evidence Synthesis (2020).

[22] Munn Z Moola S Lisy K Riitano D Tufanaru C. Methodological Guidance for Systematic Reviews of Observational Epidemiological Studies Reporting Prevalence and Cumulative Incidence Data. Int J Evid Based Healthc (2015) 13(3):147–53. 10.1097/xeb.0000000000000054 26317388

[23] Peduzzi P Concato J Feinstein AR Holford TR. Importance of Events Per Independent Variable in Proportional Hazards Regression Analysis II. Accuracy and Precision of Regression Estimates. J Clin Epidemiol (1995) 48(12):1503–10. 10.1016/0895-4356(95)00048-8 8543964

[24] Lipshutz G Baxter-Lowe LA Nguyen T Jones KD Ascher NL Feng S. Death from Donor-Transmitted Malignancy Despite Emergency Liver Retransplantation. Liver Transplant (2003) 9(10):1102–7. 10.1053/jlts.2003.50174 14526407

[25] Petit B Le Meur Y Jaccard A Paraf Fo. Robert CL Bordessoule D Influence of Host-Recipient Origin on Clinical Aspects of Posttransplantation Lymphoproliferative Disorders in Kidney Transplantation. Transplantation (2002) 73(2):265–71. 10.1097/00007890-200201270-00020 11821742

[26] Olagne J Caillard S Gaub MP Chenard MP Moulin B. Post-transplant Lymphoproliferative Disorders: Determination of Donor/recipient Origin in a Large Cohort of Kidney Recipients. Am J Transpl (2011) 11(6):1260–9. 10.1111/j.1600-6143.2011.03544.x 21564528

[27] Eccher A Girolami I Motter JD Marletta S Gambaro G Momo REN Donor-transmitted Cancer in Kidney Transplant Recipients: a Systematic Review. J Nephrol (2020) 33:1321–32. 10.1007/s40620-020-00775-4 32535833 PMC7701067

[28] The Transplantation Society of Australia and New Zealand. Clinical Guidelines for Organ Transplantation from Deceased Donors (2014). Available at: https://tsanz.com.au/guidelinesethics-documents/organallocationguidelines.htm (Accessed May 2021).

[29] Begum R Harnois D Satyanarayana R Krishna M Halling KC Kim GP Retransplantation for Donor-Derived Neuroendocrine Tumor. Liver Transpl (2011) 17(1):83–7. 10.1002/lt.22196 21254348

[30] Mrzljak A Kocman B Skrtic A Furac I Popic J Franusic L Liver Re-transplantation for Donor-Derived Neuroendocrine Tumor: A Case Report. World J Clin Cases (2019) 7(18):2794–801. 10.12998/wjcc.v7.i18.2794 31616694 PMC6789388

[31] Snape K Izatt L Ross P Ellis D Mann K O'Grady J. Donor-transmitted Malignancy Confirmed by Quantitative Fluorescence Polymerase Chain Reaction Genotype Analysis: a Rare Indication for Liver Retransplantation. Liver Transpl (2008) 14(2):155–8. 10.1002/lt.21347 18236388

[32] Loosen SH Schmeding M Roderburg C Binnebösel M Temizel I Mottaghy FM A Liver Nodule in a Patient Transplanted for Primary Sclerosing Cholangitis: an Interdisciplinary Diagnostic Approach. Z Gastroenterol (2017) 55(1):56–62. 10.1055/s-0042-111048 27706546

[33] Backes AN Tannuri ACA de Mello ES Gibelli NEM de Castro Andrade W Tannuri U. Transmission of clear Cell Tumor in a Graft Liver from Cadaveric Donor: Case Report. Pediatr Transpl (2012) 16(8):E352–E355. 10.1111/j.1399-3046.2012.01711.x 22574830

[34] Ortiz JA Manzarbeitia C Noto KA Rothstein KD Araya VA Munoz SJ Extended Survival by Urgent Liver Retransplantation after Using a First Graft with Metastasis from Initially Unrecognized Donor Sarcoma. Am J Transpl (2005) 5(6):1559–61. 10.1111/j.1600-6143.2005.00824.x 15888069

[35] Sosin M Nassif SR Girlanda R Desai CS Satoskar R Kallakury B Isolated Peritoneal Donor-Related Plasmacytoma 3 Years after Liver Transplantation: a Case Report. Am J Transpl (2014) 14(2):472–6. 10.1111/ajt.12555 24373189

[36] Gerstenkorn C Thomusch O. Transmission of a Pancreatic Adenocarcinoma to a Renal Transplant Recipient. Clin Transpl (2003) 17(5):473–6. 10.1034/j.1399-0012.2003.00072.x 14703934

[37] Romagnoli R Martini S Giacometti R David E Martina MC D'Errico A Successful Urgent Liver Retransplantation for Donor-Transmitted Hepatocellular Carcinoma. Am J Transpl (2016) 16(6):1938–9. 10.1111/ajt.13712 26752588

[38] Florman S Bowne W Kim-Schluger L Sung MW Huang R Fotino M Unresectable Squamous Cell Carcinoma of Donor Origin Treated with Immunosuppression Withdrawal and Liver Retransplantation. Am J Transpl (2004) 4(2):278–82. 10.1046/j.1600-6143.2003.00322.x 14974952

[39] Kakar S Burgart LJ Charlton MR Saito Y Halling K Thibodeau SN. Origin of Adenocarcinoma in a Transplanted Liver Determined by Microsatellite Analysis. Hum Pathol (2002) 33(4):435–6. 10.1053/hupa.2002.124332 12055679

[40] Donovan JA Simmons FA Esrason KT Jamehdor M Busuttil RW Novak JM Donor Origin of a Posttransplant Liver Allograft Malignancy Identified by Fluorescence *In Situ* Hybridization for the Y Chromosome and DNA Genotyping. Transplantation (1997) 63(1):80–4. 10.1097/00007890-199701150-00015 9000665

