# Supplementary material for: Donor-Transmitted Cancer in Orthotopic Solid Organ Transplant Recipients: A Systematic Review
Source: Transpl Int. 2022 Feb 4;35:10092. doi: 10.3389/ti.2021.10092 (PMC8842379; doi:10.3389/ti.2021.10092)
Supplement: Supplementary file 1 [file DataSheet1.DOCX]

**Online Supplement for:**

**Donor-transmitted cancer in orthotopic solid organ transplant recipients: a systematic review**

**Greenhall et al.**

**Contents**

[1 List of tables 2](#_Toc89080803)

[2 List of figures 2](#_Toc89080804)

[3 Search strategy 3](#_Toc89080805)

[3.1 Sources 3](#_Toc89080806)

[3.2 Search terms 3](#_Toc89080807)

[4 Data fields extracted 8](#_Toc89080808)

[5 Tables 9](#_Toc89080809)

[6 Figures 14](#_Toc89080810)

[7 References 17](#_Toc89080811)

# List of tables

[Table S1. Full list of articles included in review](#_Toc89080726)

[Table S2. Completeness of all data fields extracted](#_Toc89080727)

[Table S3. Presentation and outcome of all cases included in review, by histological type](#_Toc89080728)

# List of figures

[Figure S1. Quality grading of case reports](#_Toc89080747)

[Figure S2. Quality grading of registry studies](#_Toc89080748)

[Figure S3. Kaplan-Meier plot of patient survival after donor-transmitted cancer diagnosis, by tumour extent at diagnosis](#_Toc89080749)

# Search strategy

## Sources

### Indexed collections

MEDLINE (OvidSP, 1946 to present)

PubMed (e-publications ahead of print only)

Embase (OvidSP, 1974 to present)

Web of Science Core Collection (Thomson Reuters)

Scopus (Elsevier)

### Grey literature

Open Dissertations (EBSCOHost): <https://biblioboard.com/opendissertations/>

ETHOS repository of PhD theses: <https://ethos.bl.uk/AdvancedSearch.do?new=1>

WHO NOTIFY library: <https://www.notifylibrary.org/notifylibrary/search/incident>

BTS congress abstracts 1972 to 2019: <https://bts.org.uk/events-meetings/congress-abstracts/>

## Search terms

**MEDLINE**

1. Transplantation/ or Organ Transplantation/ or exp Heart Transplantation/ or Liver Transplantation/ or exp Lung Transplantation/

2. ((transplant* or retransplant* or re-transplant* or homotransplant* or allograft* or graft* or recipient*) adj5 (organ* or multiorgan* or multi-organ* or liver* or hepatic or heart* or cardiac or lung* or intestin* or bowel* or orthotopic*)).tw,kf.

3. (((transplant* or retransplant* or re-transplant* or homotransplant* or allograft* or graft*) adj3 recipient*) and organ*).tw,kf.

4. 1 or 2 or 3

5. exp Neoplasms/

6. (neoplas* or malignan* or tumo?r* or cancer* or carcinoma*).tw,kf.

7. 5 or 6

8. Tissue Donors/

9. ((donor* or donat*) adj3 (transmi* or deriv* or origin* or procur* or related or acquired or transfer* or organ*)).tw,kf.

10. 8 or 9

11. 4 and 7 and 10

12. ((donor* or donat* or transplant*) and (transmi* or deriv* or origin*)).ti. and exp *Neoplasms/ and ((transplant* or retransplant* or re-transplant* or homotransplant* or allograft* or graft*) adj5 (organ* or multiorgan* or multi-organ* or liver* or hepatic or heart* or cardiac or lung* or intestin* or bowel* or orthotopic*)).tw,kf.

13. ((((donor* or donat* or transplant*) and (transmi* or deriv* or origin*)) or ((accident* or inadvertent* or uninten*) and trans*)) and (neoplas* or malignan* or tumo?r* or cancer* or carcinoma*)).ti.

14. *Neoplasm Transplantation/

15. 4 and 14

16. 11 or 12 or 13 or 15

17. (bone marrow transplant* or stem cell transplant* or (h?ematopoietic adj2 transplant*)).ti.

18. animals/ not (humans/ and animals/)

19. 16 not (17 or 18)

20. exp cohort studies/

21. cohort*.tw.

22. controlled clinical trial.pt.

23. epidemiologic methods/

24. limit 23 to yr="1860 - 1989"

25. exp case-control studies/

26. (case* and (control* or series)).tw.

27. (case reports or letter).pt.

28. (case* adj2 (report* or stud* or histor*)).tw.

29. or/20-28

30. 19 and 29

**PubMed**

#1 ((transplant OR transplants OR transplantation* OR transplanting OR retransplant* OR re-transplant* OR homotransplant* OR allograft* OR graft* OR recipient*) AND (organ OR organs OR multiorgan* OR multi-organ* OR liver* OR hepatic OR heart* OR cardiac OR lung OR lungs OR intestin* OR bowel* OR orthotopic*))

#2 ((transplant OR transplants OR transplantation* OR transplanting OR retransplant* OR re-transplant* OR homotransplant* OR allograft* OR graft*) AND recipient* AND organ*)

#3 #1 OR #2

#4 (neoplas* OR malignan* OR tumor OR tumors OR tumour* OR cancer OR cancers OR cancerous OR carcinoma*)

#5 ((donor* OR donat*) AND (deriv* OR origin* OR related OR acquired OR procur* OR transmi* OR transfer* OR organ OR organs))

#6 #3 AND #4 AND #5

#7 ((donor*[TI] OR donat*[TI] OR transplant*[TI]) AND (transmi*[TI] OR deriv*[TI] OR origin*[TI]) AND ((transplant OR transplants OR transplantation* OR transplanting OR retransplant* OR re-transplant* OR homotransplant* OR allograft* OR graft*) AND (organ OR organs OR multiorgan* OR multi-organ* OR liver* OR hepatic OR heart* OR cardiac OR lung OR lungs OR intestin* OR bowel* OR orthotopic*))

#8 ((((donor*[TI] OR donat*[TI] OR transplant*[TI]) AND (transmi*[TI] OR deriv*[TI] OR origin*[TI])) OR ((accident*[TI] OR inadvertent*[TI] OR uninten*[TI]) AND (transmi*[TI] OR transfer*[TI]))) AND (neoplas*[TI] OR malignan*[TI] OR tumor*[TI] OR tumour*[TI] OR cancer*[TI] OR carcinoma*[TI]))

#9 #6 or #7 or #8

#10 (("bone marrow"[TI] OR "stem cell"[TI] OR hematopoietic[TI] OR haematopoietic[TI]) AND transplant*[TI])

#11 #9 NOT #10

#12 (cohort* OR "case control" OR "case series" OR "case controlled" OR "case study" OR "case studies" OR "case report" OR "case reports" OR "case history" OR "case histories" OR "follow up" OR letter)
#13 (publisher[sb] OR inprocess[sb] OR pubmednotmedline[sb])
#14 #11 AND #12 AND #13

**Embase**

1. organ transplantation/ or heart transplantation/ or intestine transplantation/ or liver transplantation/ or lung transplantation/ or orthotopic transplantation/ or retransplantation/

2. ((transplant* or retransplant* or re-transplant* or homotransplant* or allograft* or graft*) adj5 (organ* or multiorgan or multi-organ or liver* or hepatic or heart* or cardiac or lung* or intestin* or bowel* or orthotopic*)).tw,kw.

3. (((transplant* or retransplant* or re-transplant* or homotransplant* or allograft* or graft*) adj3 recipient*) and organ*).tw,kw.

4. 1 or 2 or 3

5. exp malignant neoplasm/

6. (neoplas* or malignan* or tumo?r* or cancer* or carcinoma*).tw,kw.

7. 5 or 6

8. organ donor/ or heart donor/ or liver donor/ or lung donor/ or "cadaver donor"/

9. ((donor* or donat*) adj3 (deriv* or origin* or related or acquired or procur* or transmi* or transfer* or organ*)).tw,kw.

10. 8 or 9

11. 4 and 7 and 10

12. ((donor* or donat* or transplant*) and (transmi* or deriv* or origin*)).ti. and exp *neoplasm/ and ((transplant* or retransplant* or re-transplant* or homotransplant* or allograft* or graft*) adj5 (organ* or multiorgan* or multi-organ* or liver* or hepatic or heart* or cardiac or lung* or intestin* or bowel* or orthotopic*)).tw,kw.

13. ((((donor* or donat* or transplant*) and (transmi* or deriv* or origin*)) or ((accident* or inadvertent* or uninten*) and trans*)) and (neoplas* or malignan* or tumo?r* or cancer* or carcinoma*)).ti.

14. *cancer transplantation/

15. 4 and 14

16. 11 or 12 or 13 or 15

17. (bone marrow transplant* or stem cell transplant* or (h?ematopoietic adj2 transplant*)).ti.

18. animal experiment/ not (human experiment/ or human/)

19. 16 not (17 or 18)

20. exp cohort analysis/

21. exp longitudinal study/

22. exp prospective study/

23. exp follow up/

24. cohort*.tw.

25. exp case control study/

26. (case* and (control* or series)).tw.

27. exp case study/

28. case report/

29. (case* adj2 (report* or stud* or histor*)).tw.

30. Letter.pt.

31. or/20-30

32. 19 and 30

**Web of Science**

#1 TS=((transplant* OR retransplant* OR re-transplant* OR homotransplant* OR allograft* OR graft* OR recipient*) NEAR/5 (organ* OR multiorgan* OR multi-organ* OR liver* OR hepatic OR heart* OR cardiac OR lung* OR intestin* OR bowel* OR orthotopic*))

#2 TS=(((transplant* OR retransplant* OR re-transplant* OR homotransplant* OR allograft* OR graft*) NEAR/3 recipient*) AND organ*)

#3 #1 OR #2

#4 TS=(neoplas* OR malignan* OR tumo?r* OR cancer* OR carcinoma*)

#5 TS=((­­­­­­­­­donor* OR donat*) NEAR/3 (transmi* OR deriv* OR origin* OR procur* OR related OR acquired OR transfer* OR organ*))

#6 #3 AND #4 AND #5

#7 TI=((((donor* OR donat* OR transplant*) AND (transmi* OR deriv* OR origin*)) OR ((accident* OR inadvertent* OR uninten*) AND trans*)) AND (neoplas* OR malignan* OR tumo?r* OR cancer* OR carcinoma*))

#8 #6 OR #7
#9 TI=(bone marrow transplant* OR stem cell transplant* OR hematopoietic transplant* OR haematopoietic transplant*)

#10 #8 NOT #9
#11 TS=(cohort* OR "case control" OR "case series" OR "case controlled" OR "case study" OR "case studies" OR "case report" OR "case reports" OR "case history" OR "case histories" OR "follow up" OR letter)
#12 #10 AND #11

**Scopus**

#1 TITLE-ABS-KEY ((transplant* OR retransplant* OR re-transplant* OR homotransplant* OR allograft* OR graft* OR recipient*) W/5 (organ* OR multiorgan* OR multi-organ* OR liver* OR hepatic OR heart* OR cardiac OR lung* OR intestin* OR bowel* OR orthotopic*))

#2 TITLE-ABS-KEY (((transplant* OR retransplant* OR re-transplant* OR homotransplant* OR allograft* OR graft*) W/3 recipient*) AND organ*)

#3 #1 OR #2

#4 TITLE-ABS-KEY (neoplas* OR malignan* OR tumo?r* OR cancer* OR carcinoma*)

#5 TITLE-ABS-KEY ((­­­­­­­­­donor* OR donat*) W/3 (transmi* OR deriv* OR origin* OR procur* OR related OR acquired OR transfer* OR organ*))

#6 #3 AND #4 AND #5

#7 TITLE ((((donor* OR donat* OR transplant*) AND (transmi* OR deriv* OR origin*)) OR ((accident* OR inadvertent* OR uninten*) AND trans*)) AND (neoplas* OR malignan* OR tumo?r* OR cancer* OR carcinoma*))

#8 #6 OR #7
#9 TITLE (bone marrow transplant* OR stem cell transplant* OR hematopoietic transplant* OR haematopoietic transplant*)

#10 #8 AND NOT #9
#11 TITLE-ABS-KEY (cohort* OR "case control" OR "case series" OR "case controlled" OR "case study" OR "case studies" OR "case report" OR "case reports" OR "case history" OR "case histories" OR "follow up" OR letter)
#12 #10 AND #11

**Open Dissertations**

((transplant* or retransplant* or re-transplant* or homotransplant* or allograft* or graft* or recipient*) AND (organ* or multiorgan* or multi-organ* or liver* or hepatic or heart* or cardiac or lung* or intestin* or bowel* or orthotopic*) AND (neoplas* or malignan* or tumor* or tumour* or cancer* or carcinoma*) AND (donor* or donat* OR transmi* OR deriv* OR origin* OR procur* OR related OR acquired OR transfer* OR accident* or inadvertent* or uninten))

**ETHOS Repository**

Title: transplantation

AND

Any word: cancer

AND NOT

Title: "stem cell"

OR

Title: transplantation

AND

Any word: malignancy

AND NOT

Title: "stem cell"

OR

Title: transplantation

AND

Any word: carcinoma

AND NOT

Title: "stem cell"

**WHO NOTIFY Library**

Reference Search & Adverse Occurrence Search:

Harm to a Recipient
Organs

Free Text: neoplasm OR neoplasms OR malignant OR malignancy OR malignancies OR tumor OR tumour OR tumors OR tumours OR cancer OR carcinoma

**BTS Congress conference proceedings**

Abstract booklets

1972 to 2004: Hand searches

2005 to 2020: Free text search: “malig”, “cancer”, “oma”, “tumo”, “transmi”, “deriv”

# Data fields extracted

**Article:**

- First author
- Year
- Country
- Article type
- Total number of cases of DTC in article (orthotopic / heterotopic)

**Donor:**

- Donor age
- Donor sex
- Donor cancer diagnosed
  - no / before donor assessment / during assessment/retrieval / after transplantation of at least one organ
- Time from cancer diagnosis to donation (years)

**Recipient:**

- Recipient age at DTC diagnosis
- Recipient sex
- Transplant type
- Time from transplant to DTC diagnosis (whole months, days if <1 month)
- Mode of cancer presentation
  - symptoms / graft dysfunction (i.e. no symptoms) / surveillance investigations / retrieval/implantation biopsy / recipient post-mortem only

**Tumour:**

- Primary site (e.g. skin)
- Histology (e.g. melanoma)
- Tumours extent at diagnosis
  - Confined to graft / distant metastases / both

**Treatment:**

- Systemic chemotherapy
- External beam radiotherapy
- Other (specify)
- Immunosuppression modified (reduced / stopped / alternative agent)
- Re-transplantation
  - Time from DTC diagnosis (whole months, days if <1 month)
- Palliative management only

**Outcome:**

- Total follow-up from DTC diagnosis (whole months, days if <1 month)
- Died
- Cause of death (cancer / other)
- Cancer remission
- Cancer recurrence
  - Time from remission (whole months, days if <1 month)

# Tables

Table S1. Full list of articles included in review

| **First author** | **Year** | **Country** | **Article type** | **Total DTC cases ^a^** | **DTC cases included ^b^** | **Total donors of included cases** | **Duplicate reports (author, year) ^c^** | |
| --- | --- | --- | --- | --- | --- | --- | --- | --- |
| Armanios | 2004 | USA | CR | 2 | 1 | 1 |  | |
| Backes | 2012 | Brazil | CR | 2 | 1 | 1 | Barros 2009 | |
| Baehner | 2000 | USA | CR | 3 | 1 | 1 |  | |
| Bajaj | 2010 | USA | CR | 1 | 1 | 1 |  | |
| Baquero | 1988 | USA | CR | 3 | 1 | 1 |  | |
| Begum | 2011 | USA | CR | 1 | 1 | 1 |  | |
| Bilal | 2013 | USA | CR | 1 | 1 | 1 |  | |
| Braun-Parvez | 2010 | France | CR | 4 | 2 | 1 |  | |
| Buell | 2001 | USA | RR | 10 | 5 | 5 | Lefrancois 1987, Loh 1997, Stephens 2000, Knoop 1994 | |
| Campos | 2009 | Portugal | CR | 2 | 1 | 1 |  | |
| Cankovic | 2006 | USA | CR | 2 | 1 | 1 |  | |
| Chen | 2008 | USA | CR | 1 | 1 | 1 | Fatt 2008, Nauen 2014 | |
| de Perrot | 2003 | Canada | RR | 1 | 1 | 1 |  | |
| Desai | 2012 | UK | RR | 15 | 2 | 2 | Snape 2008 | |
| Detry | 2005 | Belgium | CR | 2 | 1 | 1 |  | |
| Detry | 1993 | Belgium | CR | 2 | 1 | 1 | Meurisse 1989, Detroz 1991, Detry 1994 | |
| Donovan | 1997 | USA | CR | 1 | 1 | 1 |  | |
| Eccher | 2019 | Italy | RR | 10 | 4 | 4 |  | |
| Elder | 1997 | Australia | CR | 3 | 1 | 1 |  | |
| Felldin | 2016 | Sweden | CR | 7 | 1 | 1 |  | |
| Florman | 2004 | USA | CR | 1 | 1 | 1 |  | |
| Foltys | 2009 | USA | CR | 2 | 1 | 1 |  | |
| Gambacorta | 1991 | Italy | CR | 1 | 1 | 1 |  | |
| Garrido | 2008 | Spain | RR | 10 | 4 | 3 |  | |
| Gerstenkorn | 2003 | UK | CR | 2 | 1 | 1 |  | |
| Harbell | 2008 | USA | CR | 4 | 1 | 1 |  | |
| Henriksen | 2013 | Denmark | CR | 4 | 1 | 1 | Thoning 2013 | |
| Ison | 2009 | USA | RR | 7 | 2 | 2 | Chen 2008, Harbell 2008 | |
| Jonas | 1996 | Germany | CR | 1 | 1 | 1 | Frank 1998 | |
| Kakar | 2002 | USA | CR | 1 | 1 | 1 |  | |
| Kashyap | 2009 | USA | RR | 1 | 1 | 1 |  | |
| Kauffman | 2002 | USA | RR | 15 | 1 | 1 | Kakar 2002, Donovan 1997, Stephens 2000, Florman 2004, Loh 1997 | |
| Kim | 2013 | USA | CR | 1 | 1 | 1 |  | |
| Kim | 2009 | USA | CR | 2 | 1 | 1 |  | |
| Knoop | 1994 | Belgium | CR | 2 | 1 | 1 |  | |
| Lefrancois | 1987 | France | CR | 3 | 1 | 1 |  | |
| Lipshutz | 2003 | USA | CR | 1 | 1 | 1 |  | |
| Loh | 1997 | USA | CR | 1 | 1 | 1 |  | |
| Loosen | 2017 | Germany | CR | 1 | 1 | 1 |  | |
| Marsh | 1987 | USA | CR | 3 | 1 | 1 |  | |
| Matser | 2018 | Netherlands | CR | 4 | 2 | 1 |  | |
| Morris-Stiff | 2004 | UK | CR | 3 | 1 | 1 |  | |
| Morse | 1990 | USA | CR | 1 | 1 | 1 |  | |
| Mrzljak | 2019 | Croatia | CR | 1 | 1 | 1 |  | |
| Ortiz | 2005 | USA | CR | 1 | 1 | 1 |  | |
| Pandanaboyana | 2016 | UK | RR | 2 | 1 | 1 |  | |
| Romagnoli | 2016 | Italy | CR | 1 | 1 | 1 |  | |
| Sack | 1997 | Germany | CR | 1 | 1 | 1 | Meyding-Lamade 1996 | |
| Sanchez-Montes | 2019 | Spain | CR | 1 | 1 | 1 |  | |
| Snape | 2008 | UK | CR | 1 | 1 | 1 | Desai 2012 | |
| Sonbol | 2019 | USA | CR | 1 | 1 | 1 | Gallegos-Orozco 2010, Paripati 2013 | |
| Sosin | 2014 | USA | CR | 1 | 1 | 1 |  | |
| Stephens | 2000 | USA | CR | 4 | 2 | 1 |  | |
| Warshawsky | 2005 | USA | CR | 1 | 1 | 1 |  | |
| Yang | 2015 | USA | CR | 1 | 1 | 1 | Sharma 2013 | |
| Zelinkova | 2012 | Netherlands | CR | 1 | 1 | 1 |  | |
| Zhang | 2019 | China | CR | 3 | 1 | 1 |  | |
| Zhao | 2012 | USA | CR | 1 | 1 | 1 |  | |
| **Total** | **-** | **-** | **-** | **165** | **73** | **69** |  | |
| DTC, donor-transmitted cancer; CR, case report; RR, registry report  Table comprises only studies with at least one case eligible for inclusion. Publications with only heterotopic SOTRs not included.  ^a^ Includes all transplant types (heterotopic and orthotopic), regardless of detail provided on each case. Excludes donor-derived cancers.  ^b^ Cases eligible for inclusion: orthotopic SOTR with cancer type, clinical presentation or management, and patient survival reported.  ^c^ Other publications of cases in included in study. | | | | | | | |  |

Table S2. Completeness of all data fields extracted

| Data field | Total cases relevant | Cases with data available |
| --- | --- | --- |
| Donor age | 73 | 53 (75%) |
| Donor sex | 73 | 56 (77%) |
| Donor history of cancer | 73 | 63 (86%) |
| Time from donor cancer diagnosis to donation | 6 | 4 (67%) |
| Recipient age | 73 | 61 (84%) |
| Recipient sex | 73 | 59 (81%) |
| Time from transplantation to cancer diagnosis | 73 | 65 (89%) |
| Mode of cancer presentation | 73 | 53 (73%) |
| Extent of cancer at diagnosis | 73 | 61 (84%) |
| Description of recipient management ^a^ | 73 | 43 (59%) |
| Cause of death | 55 | 50 (91%) |
| Survival time (at least 6 months, or to death) | 73 | 49 (67%) |
| Numbers are n (%) of DTC cases.  Includes supplementary information provided by authors  ^a^ excludes cases managed exclusively palliatively | | |

Table S3. Presentation and outcome of all cases included in review, by histological type

| Primary tumour type | Total cases | Transplant type (n) | Spread beyond allograft | Re-transplanted | Died | Reference |
| --- | --- | --- | --- | --- | --- | --- |
| Melanoma | 10 | Liver (6), Heart (2), Lung (2) | 6/10 | 0/10 | 10/10 | ^1-9^ |
| Choriocarcinoma | 7 | Liver (5), Heart (2) | 7/7 | 0/7 | 5/7 | ^8,10-14^ |
| CNS tumours |  |  |  |  |  |  |
| Glioblastoma | 4 | Liver (2), Lung (2) | 3/4 | 0/4 | 4/4 | ^15-18^ |
| Pineoblastoma | 1 | Multi-visceral ^a^ | 1/1 | 0/1 | 1/1 | ^19^ |
| Medulloblastoma | 1 | Heart | 1/1 | 0/1 | 1/1 | ^20^ |
| Ganglioglioma ^b^ | 1 | Liver | 0/1 | 0/1 | 1/1 | ^21^ |
| Genitourinary tract tumours |  |  |  |  |  |  |
| Prostate adenocarcinoma | 2 | Liver (1), Heart (1) | 1/2 | 0/2 | 1/2 | ^22,23^ |
| Renal cell carcinoma | 2 | Heart (1), Heart-lung (1) | 2/2 | 0/2 | 2/2 | ^8,24^ |
| Nephroblastoma | 1 | Lung | 1/1 | 0/1 | 1/1 | ^25^ |
| Clear cell urothelial tumour | 1 | Liver | 0/1 | 1/1 | 0/1 | ^26^ |
| Germinal cell tumour | 1 | Liver | NR | 0/1 | 1/1 | ^27^ |
| Haematological malignancies |  |  |  |  |  |  |
| Myeloma / plasmacytoma | 2 | Liver (2) | 1/2 | 1/2 | 1/2 | ^28,29^ |
| Acute myeloid leukaemia | 1 | Liver | NR | NR | 1/1 | ^30^ |
| Lymphoma |  |  |  |  |  |  |
| Anaplastic large cell lymphoma | 1 | Liver | 1/1 | 0/1 | 1/1 | ^31^ |
| B cell lymphoma | 1 | Heart | 1/1 | 0/1 | 1/1 | ^32^ |
| Non-Hodgkin lymphoma | 1 | Liver | NR | NR | 1/1 | ^30^ |
| Lymphoma NOS | 1 | Liver | NR | 0/1 | 1/1 | ^33^ |
| Neuroendocrine tumours |  |  |  |  |  |  |
| Small cell NET ^c^ | 3 | Liver (3) | 0/3 | 1/3 | 2/3 | ^34-36^ |
| Neuroendocrine tumour NOS | 2 | Liver (2) | 1/2 ^d^ | 0/2 | 2/2 | ^37,38^ |
| Atypical lung carcinoid tumour | 1 | Liver | 0/1 | 1/1 | 0/1 | ^39^ |
| Paraganglioma | 1 | Liver | 0/1 | 0/1 | 0/1 | ^40^ |
| Lung tumours |  |  |  |  |  |  |
| Adenocarcinoma | 2 | Liver (1), Heart (1) | 1/2 | 1/2 | 2/2 | ^8,41^ |
| Non-small cell carcinoma NOS | 2 | Liver (1), Lung (1) | 1/2 | 0/2 | 2/2 | ^37,42^ |
| Small cell carcinoma | 1 | Liver | NR | NR | 0/1 | ^43^ |
| Broncho-alveolar carcinoma | 1 | Lung | 0/1 | 0/1 | 1/1 | ^44^ |
| Sarcoma |  |  |  |  |  |  |
| Spindle cell sarcoma | 2 | Liver (2) | 0/2 | 1/2 | 1/2 | ^45,46^ |
| Sarcoma NOS | 2 | Liver (1), Lung (1) | NR | 0/2 | 2/2 | ^27^ |
| Intra-pulmonary synovial sarcoma | 1 | Liver | 0/1 | 0/1 | 0/1 | ^47^ |
| Angiosarcoma | 1 | Lung | 1/1 | 0/1 | 1/1 | ^48^ |
| Tumours of unknown primary site |  |  |  |  |  |  |
| Adenocarcinoma | 3 | Liver (2), Heart (1) | 1/3 | 2/3 | 1/3 | ^8,49,50^ |
| Undifferentiated carcinoma | 2 | Liver (2) | NR | 0/2 ^c^ | 2/2 | ^27,30^ |
| Squamous cell carcinoma | 1 | Liver | 0/1 | 1/1 | 0/1 | ^51^ |
| Intestinal tumours |  |  |  |  |  |  |
| Colonic adenocarcinoma | 4 | Liver (4) | 0/4 | 2/4 | 3/4 | ^52-55^ |
| Intestinal NOS | 1 | Liver | NR | NR | 1/1 | ^30^ |
| Other tumours |  |  |  |  |  |  |
| Breast adenocarcinoma | 2 | Liver (1), Lung (1) | 1/2 | 0/2 | 2/2 | ^56^ |
| Hepatocellular carcinoma | 2 | Liver (2) | 0/2 | 1/2 ^c^ | 0/2 | ^43,57^ |
| Pancreatic adenocarcinoma | 1 | Liver | 0/1 | 1/1 | 0/1 | ^58^ |
| CNS, central nervous system; NOS, not otherwise specified; NET, neuroendocrine tumour; NR, not reported.  ^a^ liver-pancreas-intestine  ^b^ transformed to high-grade neoplasm of neuroglial origin  ^c^ includes 1 small cell neuroendocrine tumour of lung origin  ^d^ not reported in one case | | | | | | |

# Figures

Figure S1. Quality grading of case reports

Quality graded using Joanna Briggs Institute critical appraisal checklist for case reports.^59^

Figure S2. Quality grading of registry studies

Quality graded using Joanna Briggs Institute critical appraisal checklist for prevalence studies.^60^

Figure S3. Kaplan-Meier plot of patient survival after donor-transmitted cancer diagnosis, by tumour extent at diagnosis


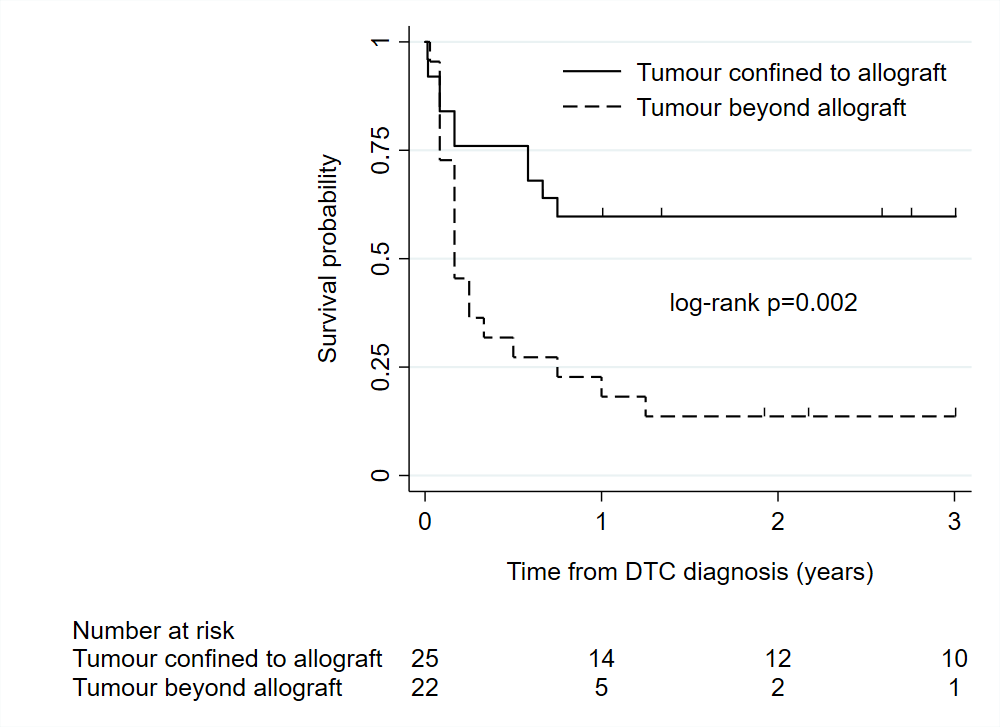


Restricted to cases with follow-up of at least six months, or to death, and extent of tumour at diagnosis reported (n=47). Follow-up censored at 3 years. DTC, donor-transmitted cancer.

# References

1. Bajaj NS, Watt C, Hadjiliadis D, et al. Donor transmission of malignant melanoma in a lung transplant recipient 32 years after curative resection. *Transpl Int.* 2010;23(7):e26-31.

2. Bilal M, Eason JD, Das K, Sylvestre PB, Dean AG, Vanatta JM. Donor-derived metastatic melanoma in a liver transplant recipient established by DNA fingerprinting. *Exp Clin Transplant.* 2013;11(5):458-463.

3. Cankovic M, Linden MD, Zarbo RJ. Use of microsatellite analysis in detection of tumor lineage as a cause of death in a liver transplant patient. *Arch Pathol Lab Med.* 2006;130(4):529-532.

4. Kim JK, Carmody IC, Cohen AJ, Loss GE. Donor transmission of malignant melanoma to a liver graft recipient: case report and literature review. *Clin Transplant.* 2009;23(4):571-574.

5. Morris-Stiff G, Steel A, Savage P, et al. Transmission of donor melanoma to multiple organ transplant recipients. *Am J Transplant.* 2004;4(3):444-446.

6. Stephens JK, Everson GT, Elliott CL, et al. Fatal transfer of malignant melanoma from multiorgan donor to four allograft recipients. *Transplantation.* 2000;70(1):232-236.

7. Warshawsky I, Farver C, Paul P, Mehta A, Decamp MM. Importance of metastatic site analysis in determining tumor lineage in a lung transplant recipient. *Transplantation.* 2005;79(7):858-859.

8. Buell JF, Trofe J, Hanaway MJ, et al. Transmission of donor cancer into cardiothoracic transplant recipients. *Surgery.* 2001;130(4):660-666.

9. Elder GJ, Hersey P, Branley P. Remission of transplanted melanoma--clinical course and tumour cell characterisation. *Clin Transplant.* 1997;11(6):565-568.

10. Braun-Parvez L, Charlin E, Caillard S, et al. Gestational choriocarcinoma transmission following multiorgan donation. *Am J Transplant.* 2010;10(11):2541-2546.

11. Detry O, Detroz B, D'Silva M, et al. Misdiagnosed malignancy in transplanted organs. *Transpl Int.* 1993;6(1):50-54.

12. Marsh JW, Jr., Esquivel CO, Makowka L, et al. Accidental transplantation of malignant tumor from a donor to multiple recipients. *Transplantation.* 1987;44(3):449-450.

13. Campos AP, Brett AC, Pinto CR, Heitor FM, Goncalves IM, Furtado ES. Acquired Choriocarcinoma in a liver graft - A case report. *Pediatr Transplant.* 2009;1):66.

14. Baquero A, Foote J, Kottle S, et al. Inadvertant transplantation of choriocarcinoma into four recipients. *Transplant Proc Journal Translated Name Transplantation Proceedings.* 1988;20(1):98-100.

15. Armanios MY, Grossman SA, Yang SC, et al. Transmission of glioblastoma multiforme following bilateral lung transplantation from an affected donor: case study and review of the literature. *Neuro Oncol.* 2004;6(3):259-263.

16. Chen H, Shah AS, Girgis RE, Grossman SA. Transmission of glioblastoma multiforme after bilateral lung transplantation. *J Clin Oncol.* 2008;26(19):3284-3285.

17. Jonas S, Bechstein WO, Lemmens HP, Neuhaus R, Thalmann U, Neuhaus P. Liver graft-transmitted glioblastoma multiforme. A case report and experience with 13 multiorgan donors suffering from primary cerebral neoplasia. *Transpl Int.* 1996;9(4):426-429.

18. Morse JH, Turcotte JG, Merion RM, Campbell DA, Jr., Burtch GD, Lucey MR. Development of a malignant tumor in a liver transplant graft procured from a donor with a cerebral neoplasm. *Transplantation.* 1990;50(5):875-877.

19. Zhao P, Strohl A, Gonzalez C, et al. Donor transmission of pineoblastoma in a two-yr-old male recipient of a multivisceral transplant: a case report. *Pediatr Transplant.* 2012;16(4):E110-114.

20. Lefrancois N, Touraine JL, Cantarovich D, et al. Transmission of medulloblastoma from cadaver donor to three organ transplant recipients. *Transplant Proc.* 1987;19(1 Pt 3):2242.

21. Kashyap R, Ryan C, Sharma R, et al. Liver grafts from donors with central nervous system tumors: a single-center perspective. *Liver Transpl.* 2009;15(10):1204-1208.

22. Loh E, Couch FJ, Hendricksen C, et al. Development of donor-derived prostate cancer in a recipient following orthotopic heart transplantation. *Jama.* 1997;277(2):133-137.

23. Sanchez-Montes C, Aguilera V, Prieto M, et al. Periesophageal Lymph Node Metastasis of Prostate Adenocarcinoma From Liver Transplant Donor. *American Journal of Gastroenterology.* 2019;114(3):378.

24. Sack FU, Lange R, Mehmanesh H, et al. Transferral of extrathoracic donor neoplasm by the cardiac allograft. *J Heart Lung Transplant.* 1997;16(3):298-301.

25. Knoop C, Jacobovitz D, Antoine M, de Francquen P, Yernault JC, Estenne M. Donor-transmitted tumors in lung allograft recipients: report on two cases. *Transplantation.* 1994;57(11):1679-1680.

26. Backes AN, Tannuri AC, de Mello ES, Gibelli NE, de Castro Andrade W, Tannuri U. Transmission of clear cell tumor in a graft liver from cadaveric donor: case report. *Pediatr Transplant.* 2012;16(8):E352-355.

27. Garrido G, Matesanz R. The Spanish National Transplant Organization (ONT) tumor registry. *Transplantation.* 2008;85(8 Suppl):S61-63.

28. Sosin M, Nassif SR, Girlanda R, et al. Isolated peritoneal donor-related plasmacytoma 3 years after liver transplantation: a case report. *Am J Transplant.* 2014;14(2):472-476.

29. Felldin M, Ekberg J, Polanska-Tamborek D, et al. Donor Monoclonal Gammopathy May Cause Lymphoproliferative Disorders in Solid Organ Transplant Recipients. *American Journal of Transplantation.* 2016;16(9):2676-2683.

30. Eccher A, Lombardini L, Girolami I, et al. How safe are organs from deceased donors with neoplasia? The results of the Italian Transplantation Network. *J Nephrol.* 2019;32(2):323-330.

31. Harbell JW, Dunn TB, Fauda M, John DG, Goldenberg AS, Teperman LW. Transmission of anaplastic large cell lymphoma via organ donation after cardiac death. *Am J Transplant.* 2008;8(1):238-244.

32. Gambacorta M, Bonacina E, Falini B, Sabattini E, Pileri S. Malignant lymphoma in the recipient of a heart transplant from a donor with malignant lymphoma. Lymphoma transplantation or de novo disease? *Transplantation.* 1991;51(4):920-922.

33. Pandanaboyana S, Longbotham D, Hostert L, et al. Transplantation of liver and kidney from donors with malignancy at the time of donation: an experience from a single centre. *Transplant Int.* 2016;29(1):73-80.

34. Baehner R, Magrane G, Balassanian R, et al. Donor origin of neuroendocrine carcinoma in 2 transplant patients determined by molecular cytogenetics. *Hum Pathol.* 2000;31(11):1425-1429.

35. Begum R, Harnois D, Satyanarayana R, et al. Retransplantation for donor-derived neuroendocrine tumor. *Liver Transpl.* 2011;17(1):83-87.

36. Foltys D, Linkermann A, Heumann A, et al. Organ recipients suffering from undifferentiated neuroendocrine small-cell carcinoma of donor origin: a case report. *Transplant Proc Journal Translated Name Transplantation Proceedings.* 2009;41(6):2639-2642.

37. Desai R, Collett D, Watson CJ, Johnson P, Evans T, Neuberger J. Cancer transmission from organ donors-unavoidable but low risk. *Transplantation.* 2012;94(12):1200-1207.

38. Kauffman HM, McBride MA, Cherikh WS, Spain PC, Hanto DW, Delmonico FL. Donor-Related Malignancies. *Transplantation Reviews.* 2002;16(4):177-191.

39. Mrzljak A, Kocman B, Skrtic A, et al. Liver re-transplantation for donor-derived neuroendocrine tumor: A case report. *World Journal of Clinical Cases.* 2019;7(18):2794-2801.

40. Yang SE, Kim C, Wang H, et al. RE: Anesthetic Management for Resection of Hepatic Paraganglioma Metastatic From the Donor Organ in an Orthotopic Liver Transplant Recipient: A Case Report. *Transplant Proc.* 2015;47(6):2072-2073.

41. Lipshutz GS, Baxter-Lowe LA, Nguyen T, Jones KD, Ascher NL, Feng S. Death from donor-transmitted malignancy despite emergency liver retransplantation. *Liver Transpl.* 2003;9(10):1102-1107.

42. Sonbol MB, Halling KC, Douglas DD, Ross HJ. A Case of Donor-Transmitted Non-Small Cell Lung Cancer After Liver Transplantation: An Unwelcome Guest. *Oncologist.* 2019;24(6):e391-e393.

43. Ison MG, Hager J, Blumberg E, et al. Donor-derived disease transmission events in the United States: data reviewed by the OPTN/UNOS Disease Transmission Advisory Committee. *Am J Transplant.* 2009;9(8):1929-1935.

44. de Perrot M, Wigle DA, Pierre AF, et al. Bronchogenic carcinoma after solid organ transplantation. *Ann Thorac Surg.* 2003;75(2):367-371.

45. Ortiz JA, Manzarbeitia C, Noto KA, et al. Extended survival by urgent liver retransplantation after using a first graft with metastasis from initially unrecognized donor sarcoma. *Am J Transplant.* 2005;5(6):1559-1561.

46. Detry O, De Roover A, de Leval L, et al. Transmission of an undiagnosed sarcoma to recipients of kidney and liver grafts procured in a non-heart beating donor. *Liver Transpl.* 2005;11(6):696-699.

47. Zhang J, Lin J. Transmission of synovial sarcoma by a single multiorgan donor to three solid organ transplant recipients: Initial report in China. *Transplantation.* 2019;103 (11 Supplement 1):S25.

48. Henriksen ISI, Ostergaard M, Talman MLM, et al. Unforeseen events: Seizures following lung transplant. *Journal of Heart and Lung Transplantation.* 2013;1):S139.

49. Donovan JA, Simmons FA, Esrason KT, et al. Donor origin of a posttransplant liver allograft malignancy identified by fluorescence in situ hybridization for the Y chromosome and DNA genotyping. *Transplantation.* 1997;63(1):80-84.

50. Kakar S, Burgart LJ, Charlton MR, Saito Y, Halling K, Thibodeau SN. Origin of adenocarcinoma in a transplanted liver determined by microsatellite analysis. *Hum Pathol.* 2002;33(4):435-436.

51. Florman S, Bowne W, Kim-Schluger L, et al. Unresectable squamous cell carcinoma of donor origin treated with immunosuppression withdrawal and liver retransplantation. *Am J Transplant.* 2004;4(2):278-282.

52. Kim B, Woreta T, Chen PH, et al. Donor-transmitted malignancy in a liver transplant recipient: a case report and review of literature. *Dig Dis Sci.* 2013;58(5):1185-1190.

53. Loosen SH, Schmeding M, Roderburg C, et al. A liver nodule in a patient transplanted for primary sclerosing cholangitis: an interdisciplinary diagnostic approach. *Z Gastroenterol.* 2017;55(1):56-62.

54. Snape K, Izatt L, Ross P, Ellis D, Mann K, O'Grady J. Donor-transmitted malignancy confirmed by quantitative fluorescence polymerase chain reaction genotype analysis: a rare indication for liver retransplantation. *Liver Transpl.* 2008;14(2):155-158.

55. Zelinkova Z, Geurts-Giele I, Verheij J, et al. Donor-transmitted metastasis of colorectal carcinoma in a transplanted liver. *Transpl Int.* 2012;25(1):e10-15.

56. Matser YAH, Terpstra ML, Nadalin S, et al. Transmission of breast cancer by a single multiorgan donor to 4 transplant recipients. *Am J Transplant.* 2018;18(7):1810-1814.

57. Romagnoli R, Martini S, Giacometti R, et al. Successful Urgent Liver Retransplantation for Donor-Transmitted Hepatocellular Carcinoma. *American Journal of Transplantation.* 2016;16(6):1938-1939.

58. Gerstenkorn C, Thomusch O. Transmission of a pancreatic adenocarcinoma to a renal transplant recipient. *Clin Transplant.* 2003;17(5):473-476.

59. Moola S, Munn Z, Tufanaru C, et al. Chapter 7: Systematic Reviews of Etiology and Risk. In: *JBI Manual for Evidence Synthesis.*2020.

60. Munn Z, Moola S, Lisy K, Riitano D, Tufanaru C. Methodological guidance for systematic reviews of observational epidemiological studies reporting prevalence and cumulative incidence data. *Int J Evid Based Healthc.* 2015;13(3):147-153.
